# 

**Published:** 1853-12

**Authors:** 


Our next number will contain articles from Drs. Meachem, Stanley, *Rusticus*, and each of the editors. We are obliged to our friends for their communications, and would suggest to others who have the leisure and ability to write, that "they go and do likewise." S. B. H.

